# Effects of crystalloid, hyper-oncotic albumin, and iso-oncotic albumin on lung and kidney damage in experimental acute lung injury

**DOI:** 10.1186/s12931-019-1115-x

**Published:** 2019-07-16

**Authors:** Renata de S. Mendes, Milena V. Oliveira, Gisele A. Padilha, Nazareth N. Rocha, Cintia L. Santos, Ligia A. Maia, Marcos V. de S. Fernandes, Fernanda F. Cruz, Priscilla C. Olsen, Vera L. Capelozzi, Marcelo Gama de Abreu, Paolo Pelosi, Patricia R. M. Rocco, Pedro L. Silva

**Affiliations:** 1 Laboratory of Pulmonary Investigation, Carlos Chagas Filho Biophysics Institute, Federal University of Rio de Janeiro, Centro de Ciências da Saúde, Avenida Carlos Chagas Filho, s/n, Bloco G-014, Ilha do Fundão, Rio de Janeiro, RJ 21941-902 Brazil; 2 Department of Physiology and Pharmacology, Biomedical Institute, Fluminense Federal University, Rio de Janeiro, Brazil; 3 Laboratory of Bacteriology and Clinical Immunology, Federal University of Rio de Janeiro, Rio de Janeiro, Brazil; 4 Department of Pathology, University of Sao Paulo, Sao Paulo, Brazil; 5 Pulmonary Engineering Group, Department of Anesthesiology and Intensive Care Therapy, University Hospital Dresden, Technische Universität Dresden, Dresden, Germany; 6 Department of Surgical Sciences and Integrated Diagnostics (DISC), University of Genoa, Genoa, Italy; 7 IRCCS San Martino Policlinico Hospital, Genoa, Italy

**Keywords:** Acute lung injury, Hemodynamic, Albumin, Inflammation, Lung damage, Kidney damage

## Abstract

**Background:**

Conflicting data have reported beneficial effects of crystalloids, hyper-oncotic albumin (20%ALB), and iso-oncotic albumin (5%ALB) in critically ill patients. Although hyper-oncotic albumin may minimize lung injury, recent studies have shown that human albumin may lead to kidney damage proportional to albumin concentration. In this context, we compared the effects of Ringer’s lactate (RL), 20%ALB, and 5%ALB, all titrated according to similar hemodynamic goals, on pulmonary function, lung and kidney histology, and molecular biology in experimental acute lung injury (ALI).

**Methods:**

Male Wistar rats received *Escherichia coli* lipopolysaccharide intratracheally (*n* = 24) to induce ALI. After 24 h, animals were anesthetized and randomly assigned to receive RL, 20%ALB, or 5%ALB (*n* = 6/group) to maintain hemodynamic stability (distensibility index of inferior vena cava < 25%, mean arterial pressure > 65 mmHg). Rats were then mechanically ventilated for 6 h. Six animals, which received neither ventilation nor fluids (NV), were used for molecular biology analyses.

**Results:**

The total fluid volume infused was higher in RL compared to 5%ALB and 20%ALB (median [interquartile range], 10.8[8.2–33.2] vs. 4.8[3.6–7.7] and 4.3[3.9–6.6] mL, respectively; *p* = 0.02 and *p* = 0.003). B-line counts on lung ultrasound (*p* < 0.0001 and *p* = 0.0002) and serum lactate levels (*p* = 0.01 and p = 0.01) were higher in RL than 5%ALB and 20%ALB. Diffuse alveolar damage score was lower in 5%ALB (10.5[8.5–12]) and 20%ALB (10.5[8.5–14]) than RL (16.5[12.5–20.5]) (*p* < 0.05 and *p* = 0.03, respectively), while acute kidney injury score was lower in 5%ALB (9.5[6.5–10]) than 20%ALB (18[15–28.5], *p* = 0.0006) and RL (16 [15–19], *p* = 0.04). In lung tissue, mRNA expression of interleukin (IL)-6 was higher in RL (59.1[10.4–129.3]) than in 5%ALB (27.0[7.8–49.7], p = 0.04) or 20%ALB (3.7[7.8–49.7], *p* = 0.03), and IL-6 protein levels were higher in RL than 5%ALB and 20%ALB (*p* = 0.026 and *p* = 0.021, respectively). In kidney tissue, mRNA expression and protein levels of kidney injury molecule (KIM)-1 were lower in 5%ALB than RL and 20%ALB, while nephronectin expression increased (*p* = 0.01 and *p* = 0.01), respectively.

**Conclusions:**

In a rat model of ALI, both iso-oncotic and hyper-oncotic albumin solutions were associated with less lung injury compared to Ringer’s lactate. However, hyper-oncotic albumin resulted in greater kidney damage than iso-oncotic albumin. This experimental study is a step towards future clinical designs.

**Electronic supplementary material:**

The online version of this article (10.1186/s12931-019-1115-x) contains supplementary material, which is available to authorized users.

## Background

Fluid replacement is often necessary in critically ill patients [1, 2]. Different fluids have different indications and contraindications in different scenarios and at different phases of the same disease [3]. Fluids must be regarded like any other drug; their management must consider the type, dose, and timing of administration [4].

In acute respiratory distress syndrome, a conservative fluid administration strategy has been associated with reduced mechanical ventilation time, less organ dysfunction, and a tendency toward reduced need for renal replacement therapy [4, 5]. Albumin is currently available as either an iso-oncotic (5%) or a hyper-oncotic (20%) solution [6]. However, conflicting data have reported beneficial effects of crystalloids, iso-oncotic albumin, and hyper-oncotic albumin in critically ill patients.

Therefore, additional experimental studies are needed to evaluate the effects of different fluids on different organs. In experimental sepsis [7] and shock resuscitation [8], hyper-oncotic albumin showed anti-inflammatory and antioxidant properties, thus minimizing lung injury. However, both in vitro [9] and in vivo studies [10] have shown that human albumin may lead to kidney damage proportional to albumin concentration. Thus, it is unknown whether iso- or hyper-oncotic human albumin concentrations may result in beneficial effects in the lungs, previously primed by endotoxin, without damaging the kidney. Based on the foregoing, we hypothesized that, compared to hyper-oncotic albumin and crystalloid solution (Ringer’s lactate, RL), iso-oncotic albumin might minimize lung and kidney damage in experimental acute lung injury (ALI). Therefore, we compared the effects of RL, 5%ALB, and 20%ALB, all titrated to reach similar hemodynamic goals, on lung function and histology, kidney histology and renal cell regeneration, expression of genes associated with lung and kidney inflammation, and endothelial cell damage in experimental ALI.

## Methods

### Study approval

This study was approved by the Ethics Committee of the Carlos Chagas Filho Institute of Biophysics (CEUA 109/16), Federal University of Rio de Janeiro, Rio de Janeiro, Brazil. All animals received humane care in compliance with the “Principles of Laboratory Animal Care” formulated by the National Society for Medical Research and the *Guide for the Care and Use of Laboratory Animals* prepared by the National Academy of Sciences, USA. The present study followed the ARRIVE guidelines for reporting of animal research [11]. Animals were housed at a controlled temperature (23 °C) and controlled light–dark cycle (12–12 h), with free access to water and food.

### Animal preparation and experimental protocol

**Fig. 1:**
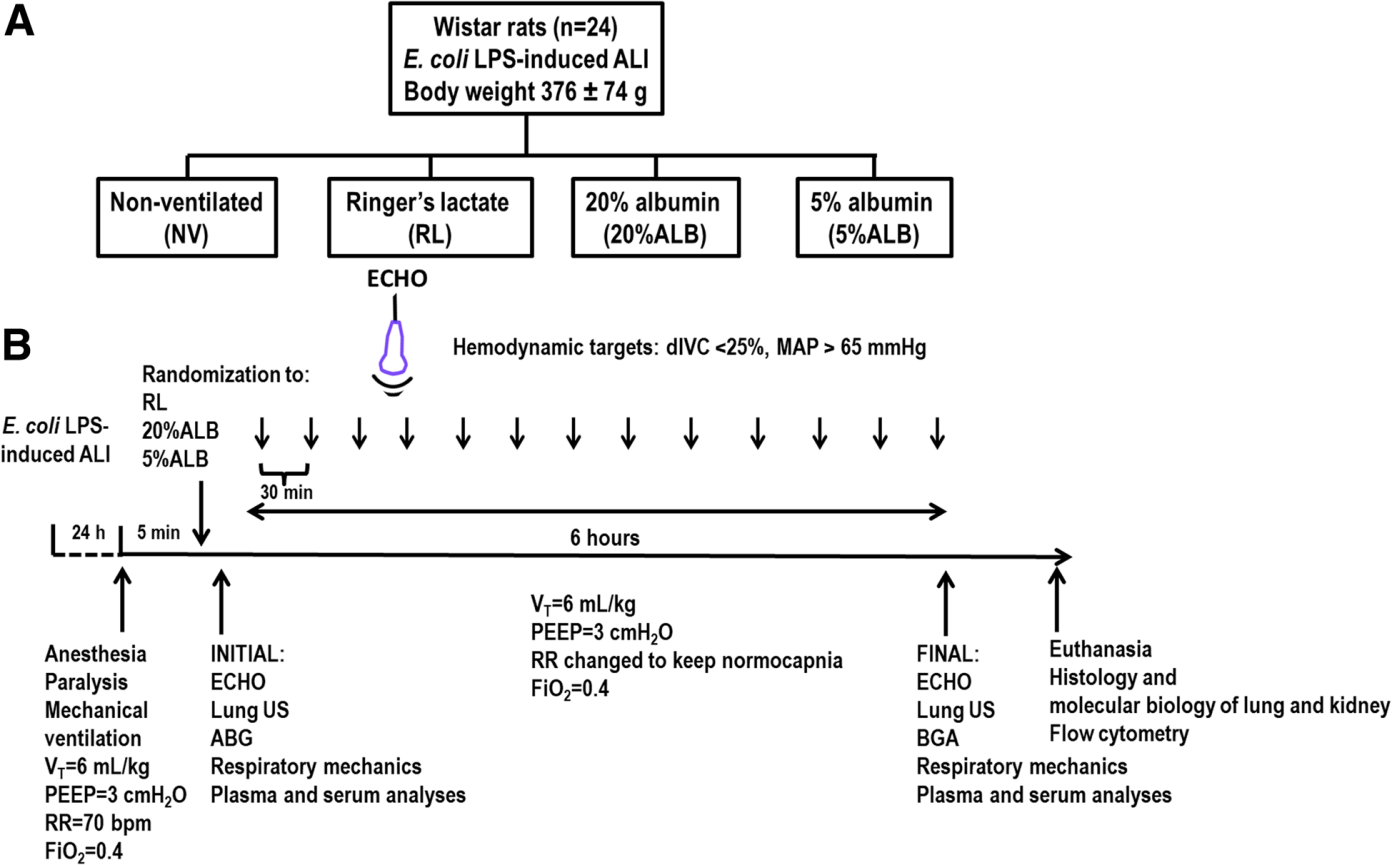
Schematic flowchart of study design (**a**) and time course of the experimental protocol (**b**). LPS, lipopolysaccharide; ALI, acute lung injury; NV, non-ventilated animals; RL, Ringer’s lactate; ALB, albumin; ECHO, echocardiography; dIVC, distensibility index of the inferior vena cava; MAP, mean arterial pressure; V_T_, tidal volume; PEEP, positive end-expiratory pressure; RR, respiratory rate; FiO_2_, fraction of inspired oxygen; US, ultrasound; BGA, blood gas analysis

Twenty-four male Wistar rats (weight 376 ± 74 g) were premedicated intraperitoneally with 4 mg/kg diazepam (Compaz®; Cristália, Itapira, SP, Brazil) and were anesthetized with 2% sevoflurane (Sevorane®; Cristália, Itapira, São Paulo, Brazil). Then, they received *Escherichia coli* lipopolysaccharide (200 μg) intratracheally (LPS-B5, serotype O55:B5, 400 μg, Invitrogen, San Diego, California, USA) to induce mild-to-moderate ALI and 25 mg/kg tramadol subcutaneously (Tramal®, Grunenthal do Brasil Farmacêutica Ltda.) for analgesia [12]. During this time, animals were monitored for the following signs: respiratory distress, hunched posture, lethargy, abnormal neurologic movements, and inability to rise from recumbency. The concurrent presence of at least two of the former signs [2] was enough to prompt immediate euthanasia by overdose of sodium thiopental (60 mg/kg) [3]. After 24 h, animals were premedicated intraperitoneally with 100 mg/kg esketamine (Ketamin-S +®; Cristália, Itapira, SP, Brazil) and 2 mg/kg midazolam (Dormicum, União Química, São Paulo, SP, Brazil). After local anesthesia with 2% lidocaine (0.4 mL), a midline neck incision and tracheostomy were performed. An intravenous (i.v.) catheter (Jelco® 24G, Becton, Dickinson and Company, USA) was inserted into the tail vein, and anesthesia induced and maintained with midazolam (2 mg/kg/h) and ketamine (50 mg/kg/h). The adequacy of anesthesia was assessed by response to a nociceptive stimulus before surgery. A second catheter (18G; Arrow International, USA) was then placed in the right internal carotid artery for blood sampling and arterial blood gas analysis (ABL80 FLEX; Radiometer Medical, Denmark), as well as monitoring of mean arterial pressure (MAP) by a liquid transducer (Utah Medical Products, Inc., Midvale, Utah, USA), and connected to a multiparameter monitor (Networked Multiparameter Veterinary Monitor LifeWindow 6000 V; Digicare Animal Health, Florida, USA). Body temperature was maintained at 37.5 ± 1 °C using a heating bed. A 30-cm-long water-filled catheter (PE-205, Becton, Dickinson and Company) with side holes at the tip, connected to a differential pressure transducer (UT-PL-400, SCIREQ, Montreal, QC, Canada), was used to measure the esophageal pressure (P_es_). The catheter was passed into the stomach and then slowly returned into the esophagus; its proper positioning was assessed with the “occlusion test” [4]. Briefly, this method consists of comparing the variation between P_es_ and tracheal pressure during spontaneous inspiratory efforts made against a closed airway. When the changes in P_es_ and tracheal pressure are comparable (difference of 5% and phase angle close to nil), this indicates that the changes in P_es_ provide a valid measure of changes in pleural pressure. Phase differences between variations of P_es_ and tracheal pressure were computed from the loop by dividing the vertical width of each loop at the midpoint of horizontal deflection by the total swing in P_es_. Animals were then paralyzed with 2 mg/kg pancuronium bromide (Pancuron®, Cristália, Itapira, SP, Brazil) and mechanically ventilated (Servo-i, MAQUET, Switzerland) in volume-controlled mode with V_T_ = 6 mL/kg, respiratory rate (RR) to keep normocapnia (PaCO_2_ = 35–45 mmHg), fraction of inspired oxygen (FiO_2_) = 0.4, and positive end-expiratory pressure (PEEP) = 3 cmH_2_O. Rats were then randomly assigned to one of three fluid groups (*n* = 6/each) (Fig. 1a): RL, 5%ALB, or 20%ALB (ALBUREX®, CSL Behring AG, Bern, Switzerland). Detailed descriptions of the composition of each fluid are provided in Additional file 1: Table S1. Functional data were obtained immediately after randomization (INITIAL) and at the end of the experiment, i.e., after 6 h of mechanical ventilation (FINAL). An additional six animals, neither ventilated nor administered fluids, were used for molecular biology analyses (group NV). At the end of the experiments, heparin (1000 IU) was injected into the tail vein and animals were euthanized by overdose of sodium thiopental (60 mg/kg). The lungs were extracted at PEEP = 3 cmH_2_O for histology and cryopreserved for further molecular biology analysis (Fig. 1b).

### Maintenance of hemodynamic stability

During mechanical ventilation, hemodynamic stability was maintained by checking the distensibility index of the inferior vena cava (dIVC) and MAP. The dIVC was measured every 30 min, while MAP was monitored continuously. Our group has validated the dIVC for small animals [13]; fluid infusion (5 mL/kg/h) should start at dIVC values > 25%, even when MAP exceeds 65 mmHg, and stop at lower dIVC values. However, when dIVC values fell below 25%, and MAP remained < 65 mmHg, a fluid bolus was infused (2 mL/kg). In short, the hemodynamic targets were dIVC < 25% and MAP > 65 mmHg.

### Transthoracic echocardiography

Transthoracic echocardiography was performed by a single experienced examiner (N.N.R.), blinded to group allocation, using an UGEO HM70A ultrasound system (Samsung, São Paulo, Brazil) equipped with a linear phased array transducer (8–13 MHz). A subcostal longitudinal view was used to visualize the inferior vena cava, performed in M-mode just upstream of the suprahepatic vein for better interpretation of maximal (Max) and minimal (Min) diameter. Max and Min measurements were obtained on the same screen. The dIVC was calculated as (Max on inspiration − Min on expiration) / Min on expiration. Immediately after each evaluation of the dIVC, the same transducer was moved to the left anterior chest wall for quantification of the left ventricular stroke volume (SV), obtained by multiplying the cross-section area of the aorta measured in B-mode parasternal long axis view of the heart by its velocity–time integral (VTI). For calculation of VTI, the pulsed-wave Doppler technique was applied in the left ventricular outflow tract [6, 7]. We also evaluated left ventricular internal diastolic diameter (LVIDD).

### Lung ultrasound

Lung ultrasound (LUS) images were obtained using the same equipment described above. The “28-rib space” technique was modified as necessary for the rat chest. LUS consisted of bilateral scanning of the anterior chest wall. Because the rat heart is close to the anterior chest wall and the heartbeat can interfere with LUS image quality, each hemithorax was divided into two quadrants: an anterior-axillary superior zone and an anterior-axillary inferior zone. Four scans were performed in each rat, always in the longitudinal plane. For each scan, A-lines and B-lines were recorded at INITIAL and FINAL. A-lines consist of repetitive horizontal artifacts parallel to the pleural line, caused by the preponderance of air over liquid in the lung parenchyma. The presence of A-lines was given a score of zero, indicating a normal LUS pattern. B-lines are defined as discrete, laser-like, vertical hyperechoic reverberation artifacts that arise from the pleural line (previously described as “comet tails”), extend to the bottom of the screen without fading, and move synchronously with lung sliding. The number of B lines was counted (ranging from 0 to 10, the latter corresponding to signal confluence) [8, 9].

### Albumin and lactate

Serum lactate (Enzychrom™ D-Lactate Assay Kit – EDLC-100, Hayward, CA, USA) was measured at INITIAL and FINAL, while plasma albumin (Albumin-PP, Gold Analisa Diagnóstica Ltda, Minas Gerais, Brazil) was evaluated only at FINAL.

### Erythrocyte apoptosis

Detection of erythrocyte apoptosis with annexin V conjugates by flow cytometry (Annexin V conjugate, ThermoFisher scientific) was performed in a BD FACSCalibur™ system (BD Biosciences, San Jose, CA). Erythrocytes previously treated with ionomycin from *Streptomyces conglobatus* (Sigma-Aldrich) were used as a positive control for erythrocyte apoptosis. Erythrocytes derived from healthy animal blood with and without annexin were used as negative controls.

### Histology

#### Diffuse alveolar damage

After euthanasia, the lungs were removed en bloc. The left lung was frozen in liquid nitrogen and immersed in formaldehyde solution (4%), embedded in paraffin, cut longitudinally in the central zone with a microtome into slices 4 μm thick, and stained with hematoxylin–eosin for histologic analysis. Photomicrographs at magnifications of × 100, × 200, and × 400 were obtained from eight non-overlapping fields of view per section using a light microscope (Olympus BX51; Olympus Latin America Inc., Brazil). Diffuse alveolar damage was quantified by two investigators (V.M. and V.L.C.) blinded to group assignment and independently, using a weighted scoring system, as described elsewhere [10]. Briefly, scores of 0 to 4 were used to represent edema, atelectasis and overdistension, with 0 standing for no effect and 4 for maximum severity. Additionally, the extent of each scored characteristic per field of view was determined on a scale of 0 to 4, with 0 standing for no visible evidence and 4 for complete involvement. Scores were calculated as the product of severity and extent of each feature, on a range of 0 to 16. The cumulative diffuse alveolar damage score was calculated as the sum of each score, and thus ranged from 0 to 48 [3].

#### Perivascular edema

To quantify perivascular edema, 10 random, non-coincident microscopic fields containing venules were evaluated. The number of points falling on areas of perivascular edema and the number of intercepts between the lines of the integrating eyepiece and the basal membrane of the vessels were counted. The interstitial perivascular edema index was calculated as follows: number of points^1/2^ ∕ number of intercepts [14].

#### Acute kidney injury (AKI) score

The left kidney was fixed in 4% buffered formaldehyde solution and embedded in paraffin. Following that, typical histological features of kidney damage [12], such as tubular necrosis, interstitial edema, and hydropic degeneration, were evaluated with a scoring system (AKI score). This score assessed involvement and severity but was slightly modified to include a weighing system for the features of interest. Briefly, values from 0 to 4 represented the severity of the feature, as follows: 0 – normal appearance, 1 – minimal injury, 2 – mild damage, 3 – moderate damage, 4 – marked damage. An additional score of 0 to 4 was used to describe the extent of involvement in each field of view, as follows: 0 – no involvement, 1 – up to 25% of tissue affected, 2–25 to 50% of tissue affected, 3–50 to 75% of tissue affected, and 4–75 to 100% of tissue affected. For each feature of interest, severity was multiplied by the extent, leading to values in the range of 0–16 for each feature; the sum score of all features was then calculated, for a maximum possible cumulative score of 48.

#### Biological markers in lung and kidney tissue

Quantitative real-time reverse transcription polymerase chain reaction (RT-PCR) was performed to measure biomarkers in lung and kidney tissue. In the lung, gene expression of biomarkers associated with inflammation (interleukin [IL]-6), endothelial integrity (VE-cadherin), and fibrosis (type III procollagen [PCIII]) was evaluated. In the kidney, gene expression of biomarkers associated with renal injury (kidney injury molecule [KIM]-1) and regeneration (nephronectin, NPNT) were evaluated. The primers are shown in Additional file 2: Table S2. For each sample, the expression of each gene was normalized to the acidic ribosomal phosphoprotein P0 (*36B4*) housekeeping gene [15] and expressed as fold change relative to NV animals, using the 2^–ΔΔCt^ method, where ΔCt = Ct_target gene_ − Ct_reference gene_ [16].

#### Enzyme-linked immunosorbent assay (ELISA)

Protein levels of IL-6 in lung tissue and KIM-1 in kidney tissue were quantified by ELISA. All procedures were done according to the manufacturer’s protocol (PeproTech, London, UK) and normalized to total protein as assessed by Bradford’s reagent (Sigma-Aldrich, St Louis, MO, USA).

### Statistical analysis

Six animals per group would provide the appropriate power (1 − β = 0.8) to identify significant (α < 0.05) differences in diffuse alveolar damage score (the primary endpoint) between RL and 25%ALB, according to previous studies [8] and pilot studies using 20%ALB, taking into account an effect size *d* = 2.02, a two-sided test, and a sample size ratio = 1 (G*Power 3.1.9.2; University of Düsseldorf, Düsseldorf, Germany).

Data were tested for normality using the Kolmogorov–Smirnov test with Lilliefors’ correction, while the Levene median test was used to evaluate the homogeneity of variances. If both conditions were satisfied, Mauchly’s test of sphericity with repeated-measures ANOVA (*p* < 0.05) was used. If epsilon was > 0.75, the Huynh–Feldt *p*-value was shown; otherwise, the Greenhouse–Geisser *p*-value was provided. Additionally, to compare hemodynamic parameters among groups at each time point, a mixed linear model based on a random intercept for each animal followed by Bonferroni’s test was used.

Functional data obtained at INITIAL and FINAL were assessed by two-way ANOVA followed by Holm–Šídák’s multiple comparisons to compare parameters among groups and between time (INITIAL and FINAL). Molecular biology variables were compared using the Kruskal–Wallis test followed by Dunn’s multiple comparisons. Parametric data were expressed as mean ± SD, and nonparametric data, as median (interquartile range). The mixed linear models and Mauchly’s sphericity tests were performed using IBM SPSS Statistics for Windows, Version 19.0 (IBM Corp., Armonk, NY, USA). All other tests were performed in GraphPad Prism version 6.00 (GraphPad Software, La Jolla, CA, USA). Significance was established at *p* < 0.05.

## Results

### Effects of different fluids on hemodynamics, serum lactate, and serum albumin

At FINAL, dIVC was < 25% regardless of fluid (Table 1), with MAP > 65 mmHg. MAP was higher in 20%ALB compared to RL animals (*p* = 0.04). Serum lactate levels and total volume infused were higher in RL than in both ALB groups (*p* = 0.01, p = 0.01; respectively). Over time, lactate levels increased only in the RL group, which may suggest impairment in peripheral perfusion. Serum albumin level increased in 20%ALB, but not in 5%ALB, compared to RL (*p* = 0.002) (Table 1).

**Table 1:** Total volume infused and cardiovascular parameters

Parameter	Group	Initial	Final	*p*-value
Total volume infused (mL)	RL	–	10.9 [8.2–33.2]	–
	20%ALB	–	4.8 [3.6–7.7]^*^	
	5%ALB	–	4.3 [3.9–6.6]^*^	
MAP (mmHg)	RL	133 ± 16	85 ± 10 ^†^	0.007^a^
	20%ALB	110 ± 24	105 ± 23 ^*^	
	5%ALB	115 ± 25	112 ± 25	
HR (bpm)	RL	418 ± 66	311 ± 116	0.571^a^
	20%ALB	445 ± 150	390 ± 176	
	5%ALB	375 ± 21	311 ± 116	
dIVC (%)	RL	44.3 [36.7–82.1]	21.1 [14.0–23.7]	0.003^a^
	20%ALB	61.1 [39.4–92.6]	13.7 [4.1–24.8]	
	5%ALB	45.0 [35.0–54.2]	15.4 [11.6–20.6] ^†^	
SV (mL)	RL	0.28 ± 0.08	0.23 ± 0.07	0.586^a^
	20%ALB	0.37 ± 0.11	0.39 ± 0.04	
	5%ALB	0.32 ± 0.11	0.26 ± 0.13	
LVIDD (cm)	RL	0.33 ± 0.06	0.44 ± 0.12	0.262^a^
	20%ALB	0.44 ± 0.07	0.53 ± 0.08 ^†^	
	5%ALB	0.44 ± 0.08	0.42 ± 0.03	
Lactate (mmol/L)	RL	3.0 ± 1.3	6.4 ± 3.1 ^†^	0.022^a^
	20%ALB	2.2 ± 1.4	2.7 ± 3.1 ^*^	
	5%ALB	3.2 ± 0.6	3.0 ± 1.5 ^*^	
Albumin (mg/dL)	RL	–	0.96 [0.65–1.76]	–
	20%ALB	–	2.45 [2.23–2.65]^*^	
	5%ALB	–	2.26 [1.60–3.30]	

Data are shown as mean ± SD or median [interquartile range]. RL: Ringer’s lactate; 20%ALB: 20% albumin; 5%ALB: 5% albumin. Comparisons were done using Mauchly’s test of sphericity with repeated-measures ANOVA (p < 0.05). If epsilon was > 0.75, the Huynh-Feldt *p*-value was shown (superscript a); otherwise, the Greenhouse-Geisser *p*-value was shown (superscript b). Additionally, to compare all parameters among groups at each time point, a mixed linear model based on a random intercept for each animal followed by Bonferroni’s test. For comparison of total volume infused, the Kruskal–Wallis test was used. MAP: mean arterial pressure; HR: heart rate: dIVC: distensibility index of the inferior vena cava; SV: stroke volume; LVIDD: left ventricular internal diastolic diameter. * vs RL (*p* < 0.05); # vs 5%ALB (*p* < 0.05); ^†^ vs Initial (*p* < 0.05)

### Effects of different fluids on blood acid-base parameters

Bicarbonate decreased in RL compared to both ALB groups (*p* = 0.0002 and *p* = 0.0001, respectively). There were no differences in pHa, PaCO_2_, and PaO_2_/FiO_2_ (Additional file 3: Table S3).

### Effects of different fluids on lung ultrasonography

**Fig. 2:**
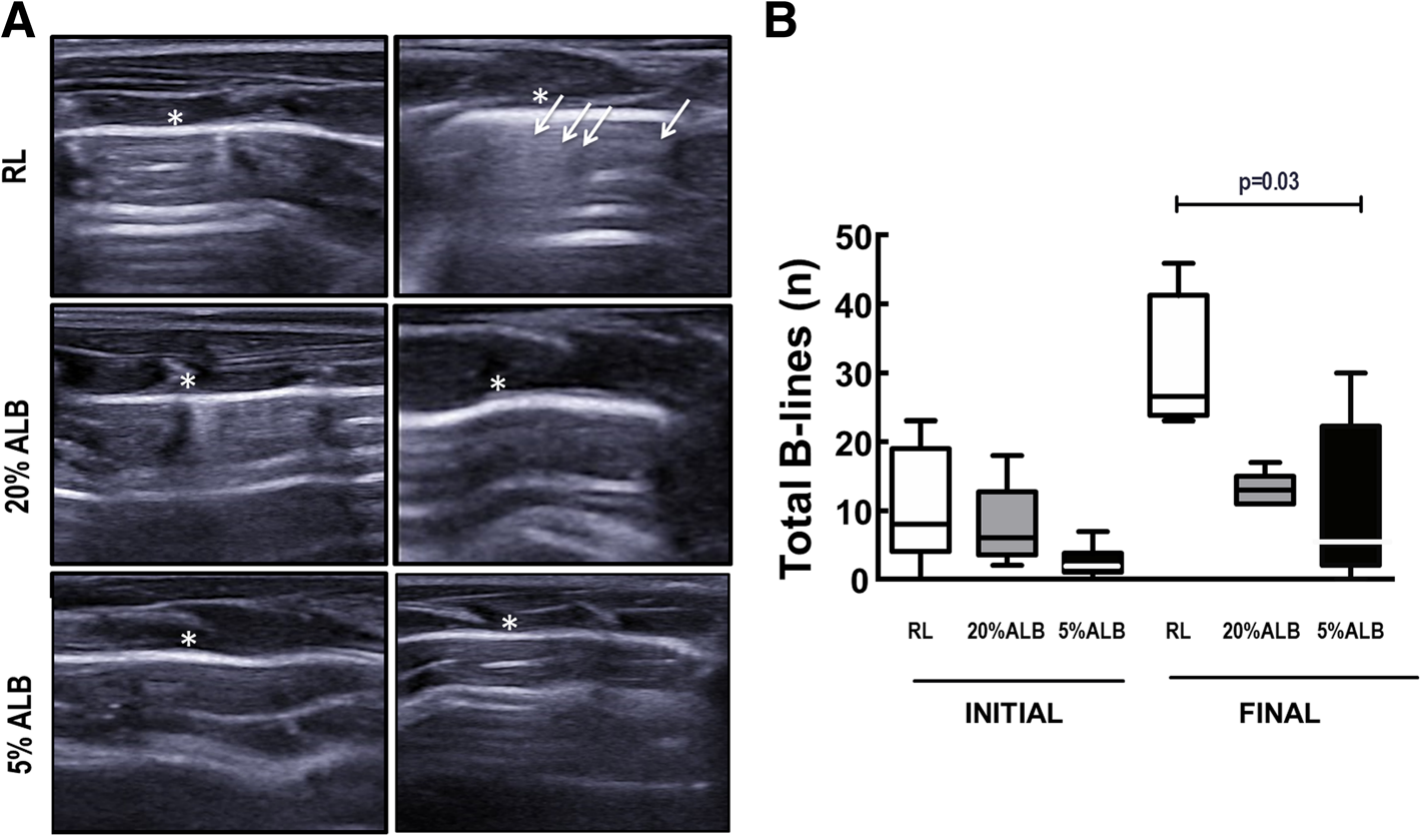
Lung ultrasound and B-lines. **a**: RL, Ringer’s lactate; ALB, albumin. B-lines (white arrows) are visible as coherent echoic bundles with a narrow base, spreading from the pleural line to the edge of the screen without fading. * Pleura. **b**: Boxes show the interquartile range (25th–75th percentile), while whiskers encompass the range (minimum-maximum) and horizontal lines represent the median in 6 animals/group. The Kruskal–Wallis test was used to compare groups at INITIAL and FINAL

B-lines, which denote lung edema, were more numerous in RL than both ALB groups at FINAL (Fig. 2).

### Effects of different fluids on lung histology

**Fig. 3:**
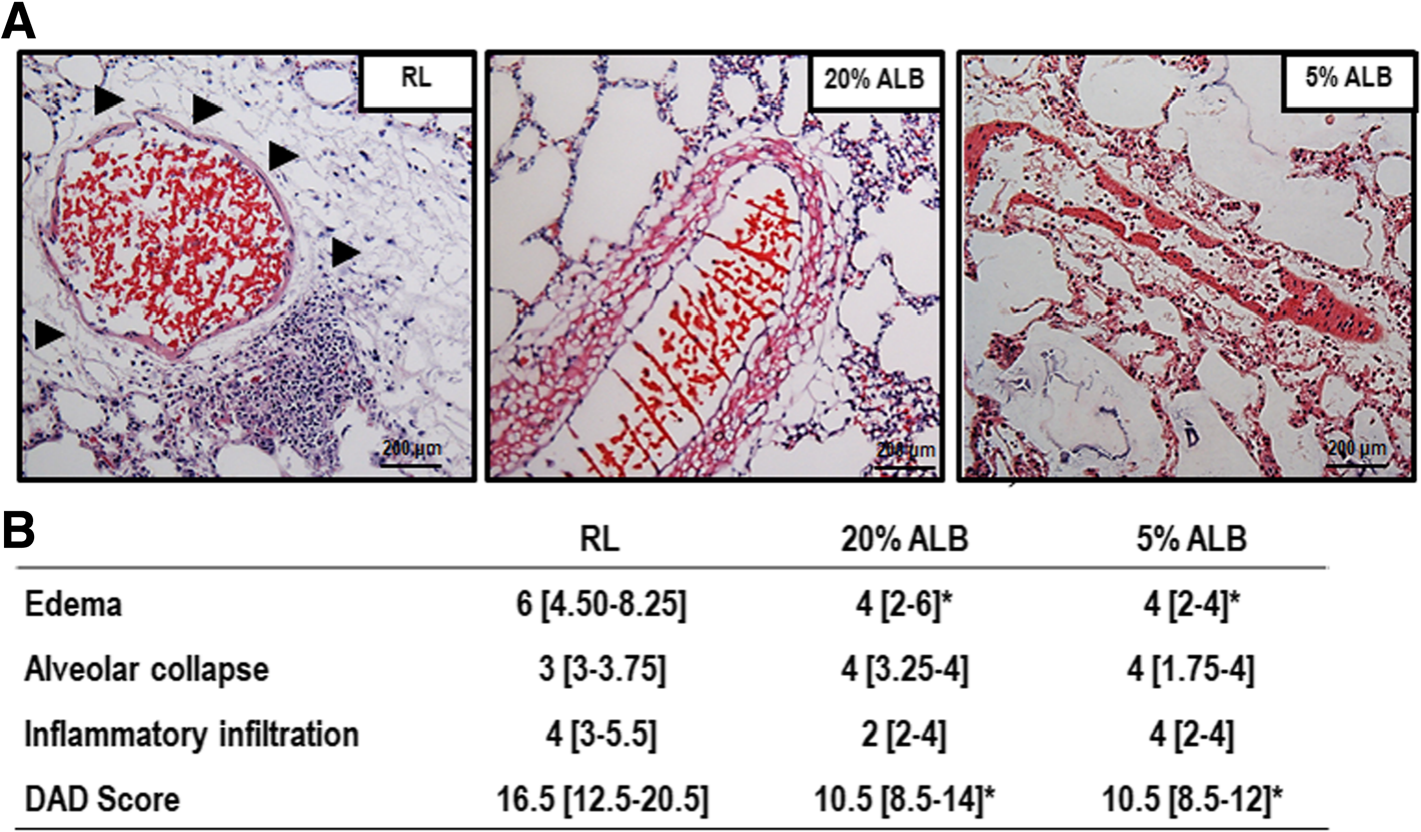
Diffuse alveolar damage (DAD) score. **a**: Representative photomicrographs (light microscopy) of lung parenchyma stained with hematoxylin and eosin. Photomicrographs are representative of data obtained from lung sections of 6 animals (original magnification, × 200). Arrows indicate perivascular edema. Bars = 200 μm. **b**: Data are shown as median [interquartile range]. The Kruskal–Wallis test was used to compare groups. * vs. RL (*p* < 0.05)

DAD score was lower in both ALB groups compared to RL, mainly due to lung edema (Fig. 3) (*p* = 0.03, *p* = 0.005 respectively). Accordingly, perivascular edema in lung tissue was lower 5%ALB and 20%ALB than RL (0.20 ± 0.02 and 0.18 ± 0.03 vs. 0.25 ± 0.04, respectively; *p* = 0.003 and *p* = 0.006).

### Effects of different fluids on lung mechanics and erythrocyte apoptosis

No major differences were observed in respiratory system and lung mechanics (Additional file 4: Table S4). Erythrocyte apoptosis, analyzed by flow cytometry, was similar across all groups.

### Effects of different fluids on biological markers associated with inflammation, endothelial cell damage, and fibrosis in lung tissue

In lung tissue, mRNA expression of IL-6, a marker of lung inflammation, was higher in RL than 5%ALB and 20%ALB (*p* = 0.04 and *p* = 0.03, respectively). Gene expression of PC-III, a marker of lung fibrogenesis, decreased in 5%ALB compared to RL and 20%ALB (*p* = 0.002 and *p* = 0.03, respectively). In addition, VE-cadherin, a marker of endothelial integrity, was higher in the 20%ALB and 5%ALB groups than in the RL group (*p* = 0.003 and *p* = 0.04, respectively) (Fig. 5). IL-6 protein levels were higher in RL (7.2 [4.5–9.2]) than in 5%ALB (1.7 [1.2–2.7], *p* = 0.026) and 20%ALB (1.8 [1.3–2.1], *p* = 0.021) (Fig. 6a).

### Effects of different fluids on kidney damage

**Fig. 4:**
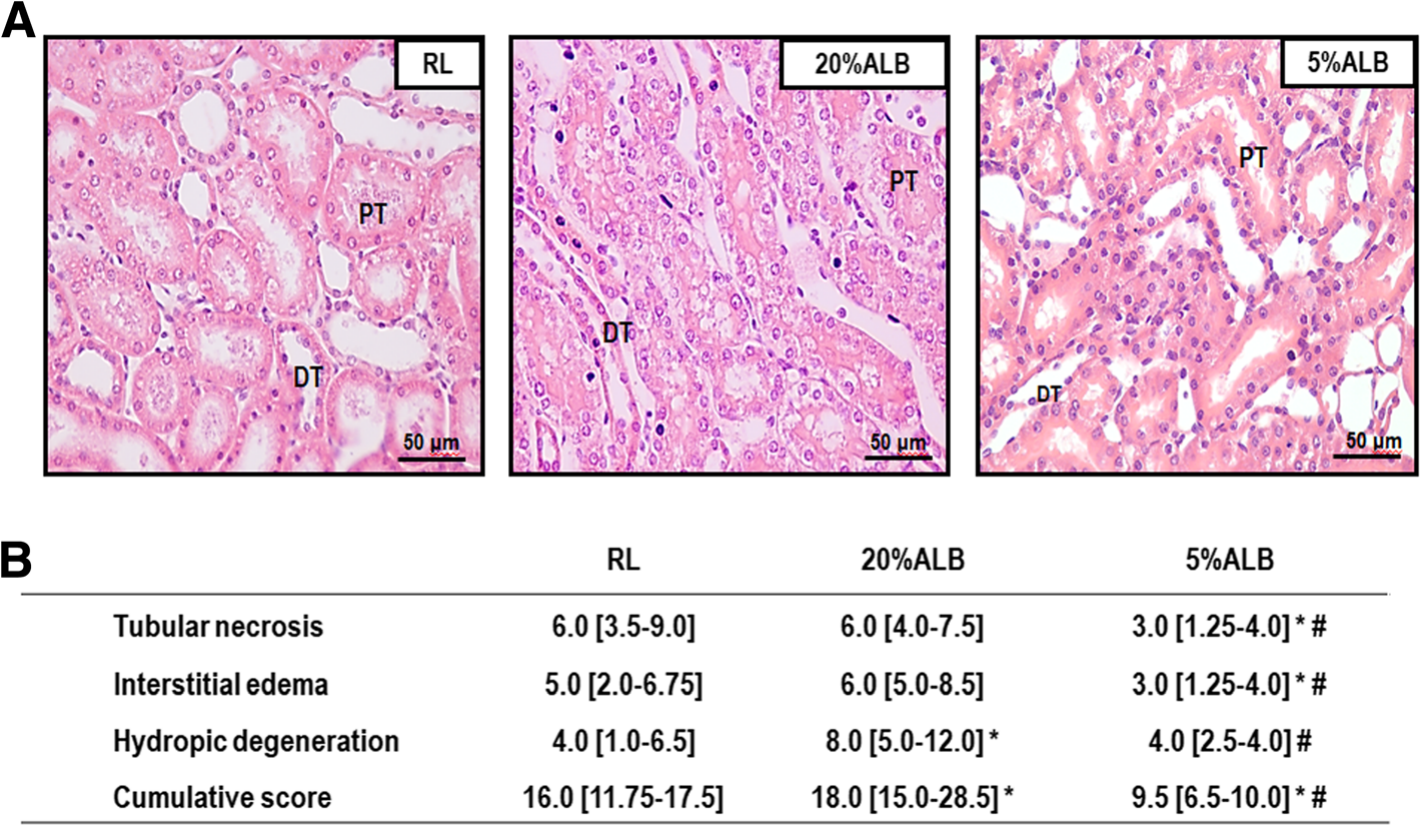
Acute kidney injury (AKI) score. **a**: Representative photomicrographs (light microscopy) of kidney parenchyma stained with hematoxylin and eosin. Photomicrographs are representative of data obtained from kidney sections of 6 animals (original magnification, × 200). PT: proximal tubule; DT: distal tubule. Bars = 50 μm. B: Data are shown as median [interquartile range]. The Kruskal–Wallis test was used to compare groups. * vs. RL (*p* < 0.05); # vs. 20%ALB (*p* < 0.05)

The AKI score was lower in 5%ALB groups compared to 20%ALB (*p* = 0.0006) and RL (p = 0.04) mainly due to reduced tubular necrosis, interstitial edema and hydropic degeneration (Fig. 4).

### Effects of different fluids on biological markers associated with kidney injury and regeneration

**Fig. 5:**
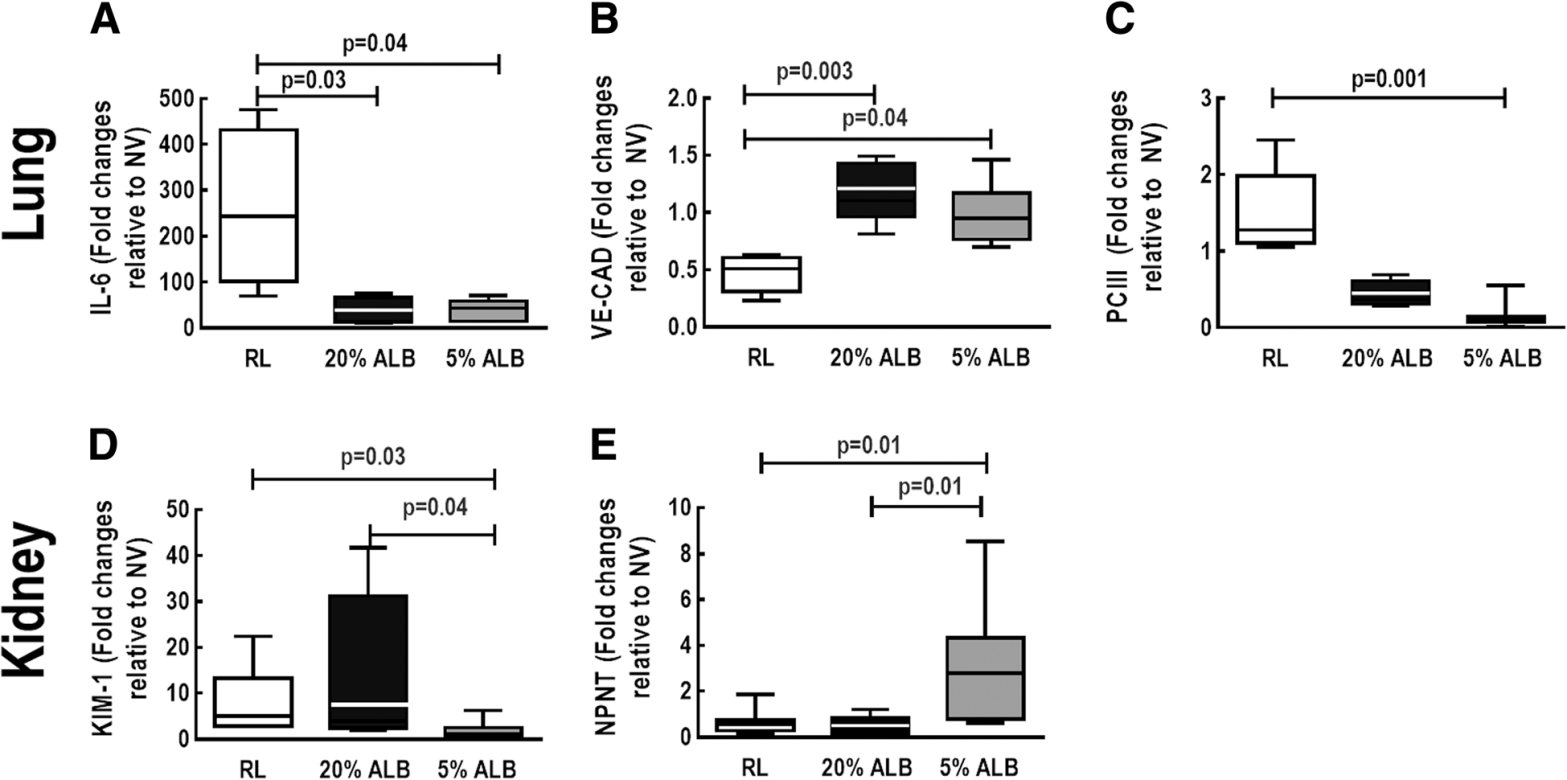
Expression of biologic markers associated with lung and kidney damage. Real-time polymerase chain reaction analysis of biologic markers associated with lung inflammation (interleukin [IL]-6) (**a**), vascular integrity (VE-CAD) (**b**), and fibrosis (procollagen [PC]-III) (**c**), as well with renal injury (kidney injury molecule [KIM-1]) (**d**) and regeneration (nephronectin, NPNT) (**e**). Boxes show the interquartile range (25th–75th percentile), while whiskers encompass the range (minimum-maximum) and horizontal lines represent the median in 6 animals/group. Relative gene expression was calculated as a ratio of average expression of each gene to the reference gene (*36B4*) and expressed as fold change relative to non-ventilated animals (NV) and no fluid. Values are medians and interquartile ranges of 6 rats in each group. Comparisons among all groups were done by the Kruskal–Wallis test followed by Dunn’s test (*p* < 0.05)

**Fig. 6:**
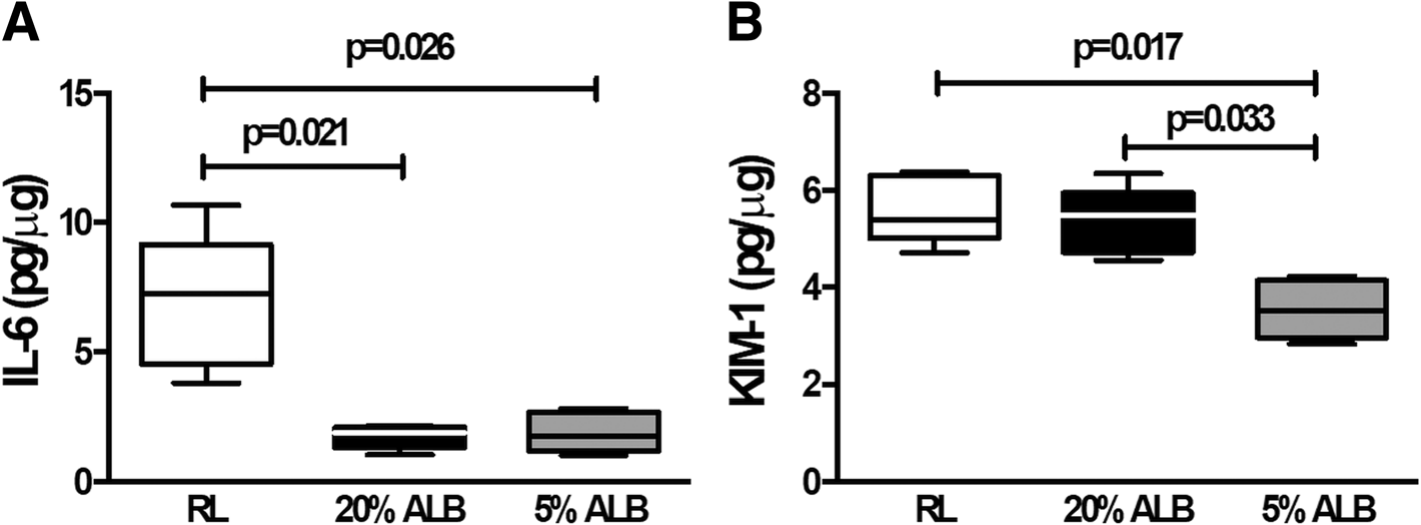
Protein levels of interleukin (IL)-6 in lung tissue (**a**) and kidney injury molecule (KIM)-1 in kidney tissue (**b**). Boxes show the interquartile range (25th–75th percentile), while whiskers encompass the range (minimum-maximum) and horizontal lines represent the median in 5 animals/group. Comparisons among all groups were done by the Kruskal–Wallis test followed by Dunn’s test (*p* < 0.05)

In kidney tissue, mRNA expression of KIM-1, an early marker of renal proximal tubular injury (17), was lower in 5%ALB than RL and 20%ALB (p = 0.03 and *p* = 0.04, respectively), while NPNT, which reflects early kidney regeneration and repair [17], was higher in 5%ALB than RL and 20%ALB (Fig. 5) (*p* = 0.01 and p = 0.01, respectively). KIM-1 protein levels were also lower in 5%ALB compared to RL and 20%ALB (*p* = 0.02 and *p* = 0.03, respectively) (Fig. 6b).

These findings suggest that iso-oncotic albumin may be associated with less kidney injury and improvement of renal-cell lesion repair.

## Discussion

In experimental ALI, both iso-oncotic and hyper-oncotic albumin, compared to Ringer’s lactate, reduced total DAD score, number of B-lines on lung ultrasound, and lung tissue inflammation, while preserving endothelial barrier integrity. However, only iso-oncotic albumin was associated with a decreased AKI score, reduced expression of KIM-1, and increased nephronectin compared with hyper-oncotic albumin and RL.

Intratracheal instillation of endotoxin induces lung alterations that resemble human acute respiratory distress syndrome (ARDS), including changes to lung mechanics and histology, and alveolar–capillary barrier injury. According to the American Society Committee Recommendations, these features characterize experimental ALI [18]. The hemodynamic stability target was based first on the dIVC (< 25%), previously described for monitoring volume status in small animals [13], and then on MAP > 65 mmHg, a target advocated by the Surviving Sepsis Campaign [19]. The dIVC was chosen since it has a linear relationship with cardiac index [20], and therefore might be useful to study the Frank-Starling curve of the heart under experimental conditions. The amount of fluids required to achieve the target dIVC was higher in RL compared to both colloid solutions, with a ratio of ~ 2.5. This has been reported elsewhere [21], and reflects the role of colloids as volume expanders. However, no difference in total amount of fluid administered was observed between iso- and hyper-oncotic solutions, despite their theoretically differing oncotic pressures. This could be associated with albumin clearance rate, which can reach 50% in rats within 6 h [22]; thus, the expected oncotic pressure differences between 5% ALB and 20% ALB may not be a constant fourfold higher rate. A similar protective ventilation strategy was maintained across groups, avoiding possible injurious effects of mechanical ventilation. Unlike in previous studies, where the focus was on individual organs, we compared the effects of RL, 5%ALB, and 20%ALB on both lung and kidney injuries, as well as on metabolic parameters related to systemic perfusion. Additionally, since the total amount of 5%ALB and 20%ALB infused was similar, a controlled evaluation of the effects of different albumin concentrations on lung and kidney injury was possible.

Regarding lung injury, both iso-oncotic and hyper-oncotic albumin compared with RL were associated with lower DAD scores (mainly due to decreased edema) and fewer B-lines on lung ultrasonography, which correlates with reduced interstitial edema [23]. B-lines have been used to quantify lung edema with good reproducibility in experimental scenario [24]. These findings may be explained by the larger volume of fluids needed to maintain hemodynamic stability with RL than with albumin solutions. As a resuscitation fluid, albumin results in a higher oncotic pressure and a short-term increase in intravascular volume. Thus, both iso- and hyper-oncotic albumin have the potential to reduce risk of lung interstitial edema [6, 25]. Furthermore, both iso- and hyper-oncotic albumin preserved the integrity of the endothelial barrier, as shown by the higher gene expression of VE-cadherin in lung tissue than seen in the RL group. This might be explained by a reduction in lung inflammation, as represented by IL-6 gene expression and protein levels. In fact, inflammatory mediators may decrease adhesive bonds between apposed endothelial cells, reducing VE-cadherin levels [26]. Our data are in line with those of a previous experimental study reporting less lung injury with hyper-oncotic albumin as compared to saline solution in a murine sepsis model [7], but differ from those of one where hyper-oncotic albumin minimized lung damage compared to iso-oncotic albumin and RL in a hemorrhagic shock model [8]. These results are likely explained by the fact that hemorrhagic shock is associated with less alveolar–capillary barrier damage than observed in sepsis-induced lung inflammation, yielding more efficient drainage of fluids from the interstitium towards the intravascular capillary compartment [27]. Moreover, iso-oncotic albumin led to decreased expression of PC-III compared to RL, suggesting that the concentration of albumin may affect the organization of extracellular matrix components, thus reducing the fibrogenic response after lung injury.

Excessive apoptosis of erythrocytes may lead to impairment of microcirculation, as they adhere to endothelial cells [28]. A wide variety of conditions stimulate erythrocyte apoptosis, such as dehydration, energy depletion, oxidative stress, and blood osmolarity [29]. In the present study, no differences in erythrocyte apoptosis were observed, which may suggest that the tested fluids do not affect microcirculation in the time frame of the experiment.

In the kidney, iso-oncotic albumin compared to hyper-oncotic albumin decreased AKI score, with specific reductions in tubular necrosis, interstitial edema, and hydropic degeneration. Hyper-oncotic albumin may result in high osmotic pressure, which may promote renal dysfunction due to altered intraglomerular oncotic forces and osmotic nephrosis [30]. Additionally, iso-oncotic albumin was associated with reduced KIM-1 and increased nephronectin expression in renal tissue compared to hyper-oncotic albumin or RL. In fact, previous in vitro [9] and in vivo [10] studies have shown detrimental effects on the kidney proportional to albumin concentrations. Thus, administration of progressively increasing albumin concentrations does not seem to be effective at minimizing acute kidney injury. This is likely explained by increased oncotic pressure in the glomerulus, which reduces ultrafiltration efficiency, as well as by the fact that higher albumin concentrations promote cell death when they reach the proximal tubules [31].

Compared to iso- and hyper-oncotic albumin, more RL was needed to maintain hemodynamic targets. This can be explained by differences in fluid composition, which may affect expansion of plasma volume. For example, albumin solutions remain in the intravascular compartment for longer than crystalloid solutions do [32]. Due to this difference in plasma volume expansion, more crystalloids were administered compared to albumin considering the same protocol of fluid administration. Furthermore, since heart rate was comparable among groups, a possible role of the sympathetic nervous system on regional perfusion and lactate production can be at least partially ruled out [33].

### Possible clinical implications

Our data may have clinical implications. RL is the most widely used crystalloid for fluid challenge and clinical treatment of critically ill patients [34]. Our data suggest that, at least in experimental ALI, RL may lead to greater lung and kidney damage than both iso- and hyper-oncotic albumin solutions. Furthermore, different albumin solutions have different impacts on peripheral organ dysfunction [8, 30]. Randomized clinical trials have shown beneficial clinical outcomes of hyper-oncotic albumin without impact on serum creatinine [35], which is a marker of kidney function but not kidney injury [36, 37]. In the present study, use of hyper-oncotic albumin solution was associated with kidney damage, according to AKI score and molecular markers of kidney dysfunction.

### Limitations

This study has limitations. First, our results may not be applied to other models of experimental ALI. Second, plasma osmolarity changes were not calculated; however the levels of serum albumin were higher in the albumin groups compared to the RL group (Additional file 1: Table S1). Third, since mechanical ventilation was limited to a duration of 6 h, our findings cannot be extrapolated to longer periods. Fourth, pH and electrolytes differ according to fluid composition [38] [39], which may affect biological markers. However, in the present study, the pH of the studied fluid solutions ranged from 6.30–7.04; moreover, considering the different volumes of fluid administration, the resulting pHa did not differ significantly (Additional file 3: Table S3).

## Conclusion

In a rat model of ALI, both iso-oncotic and hyper-oncotic albumin solutions were associated with less lung injury compared to RL. However, hyper-oncotic albumin led to greater kidney damage compared to iso-oncotic albumin. The current study is a step towards designing future clinical trials to address the impact of different concentrations of albumin solutions on different organs.

## Additional files

Additional file 1: **Table S1.** Fluid composition. (DOCX 17 kb)

Additional file 2: **Table S2.** Forward and reverse oligonucleotide sequences of target gene primers. (DOCX 21 kb)

Additional file 3: **Table S3.** Arterial blood gas analysis. (DOCX 18 kb)

Additional file 4: **Table S4.** Respiratory system and lung mechanical parameters (DOCX 21 kb)

## Data Availability

The datasets used and/or analyzed during the present study are available from the corresponding author on reasonable request.

## Abbreviations

36B4: Acidic ribosomal phosphoprotein P0
AKI: Acute kidney injury
ALB: Albumin
ALI: Acute lung injury
ARDS: Acute respiratory distress syndrome
DAD: Diffuse alveolar damage
dIVC: Distensibility index of the inferior vena cava
ELISA: Enzyme-linked immunosorbent assay
FiO_2_: Fraction of inspired oxygen
IL: Interleukin
KIM: Kidney injury molecule
LPS: Lipopolysaccharide
LUS: Lung ultrasound
LVIDD: Left ventricular internal diastolic diameter
MAP: Mean arterial pressure
NPNT: Nephronectin
NV: Not ventilated
PC: Procollagen
PEEP: Positive end-expiratory pressure
RL: Ringer’s lactate
RR: Respiratory rate
RT-PCR: Quantitative real-time reverse transcription polymerase chain reaction
SV: Stroke volume
