# Supplementary material for: Effects of crystalloid, hyper-oncotic albumin, and iso-oncotic albumin on lung and kidney damage in experimental acute lung injury
Source: Respir Res. 2019 Jul 16;20:155. doi: 10.1186/s12931-019-1115-x (PMC6636113; doi:10.1186/s12931-019-1115-x)
Supplement: Supplementary file 1 — Table S1. Fluid composition. (DOCX 17 kb) [file 12931_2019_1115_MOESM1_ESM.docx]

**Supplemental Digital Content 1**

**Supplemental Table 1:** Fluid composition

| **Composition** | **RL**  Eurofarma® | **20%ALB**  CSL Behring® | **5%ALB**  Baxalta® |
| --- | --- | --- | --- |
| **Osmolality** (mOsm/kg) | 255 | 263 | 250 |
| **Sodium caprylate** (mmol/L) | — | 16 | 4 |
| **Sodium *N*-acetyltryptophanate** (mmol/L) | — | 16 | 4 |
| **Na^+^** (mEq/L) | 130 | 140 | 140 |

| **Cl**^−^ (mEq/L) | 109.7 | — | — |
| --- | --- | --- | --- |

| **K^+^** (mEq/L) | 4 | — | — |
| --- | --- | --- | --- |
| **Ca^2+^** (mEq/L) | 2.7 | — | — |
| **pH** | 6.30 | 7.04 | 7.03 |
| **Sodium lactate** (mEq/L) | 27 | — | — |
| **Human albumin** (g/L) | — | 200 | 50 |
