# Supplementary material for: Effects of crystalloid, hyper-oncotic albumin, and iso-oncotic albumin on lung and kidney damage in experimental acute lung injury
Source: Respir Res. 2019 Jul 16;20:155. doi: 10.1186/s12931-019-1115-x (PMC6636113; doi:10.1186/s12931-019-1115-x)
Supplement: Supplementary file 2 — Table S2. Forward and reverse oligonucleotide sequences of target gene primers. (DOCX 21 kb) [file 12931_2019_1115_MOESM2_ESM.docx]

**Supplemental Digital Content 2**

**Supplemental Table 2**: Forward and reverse oligonucleotide sequences of target gene primers

| **Gene** | **Primer** | **Primer sequences (5′-3′)** |
| --- | --- | --- |
| *Lungs* |  |  |
| IL-6 | Forward | CTC CGC AAG AGA CTT CCA G |
|  | Reverse | CTC CTC TCC GGA CTT GTG A |
| PCIII | Forward | ACC TGG ACC ACA AGG ACA C |
|  | Reverse | TGG ACC CAT TTC ACC TTT C |
| VE-cadherin | Forward | CAA TAC CGC CAA CAT CAC AG |
|  | Reverse | TGG TGA GGA TGC ACA GAA AG |
| *Kidney* |  |  |
| KIM-1 | Forward | GAA GAA AAC AAT GGA TCA AGG GAT |
|  | Reverse | GGA GTG GAA ATG GCT CTA ATG AAC |
| NPNT | Forward | Gga cct gtg cct gtc ctt ta |
|  | Reverse | Tga aga tga cgc ttt tga cg |
| 36B4 | Forward | GGA TCA CTC AGG AGC AGG AG |
|  | Reverse | CTT GGC ACT CAA GAG GAA GG |

IL-6, interleukin-6; PCIII, procollagen III; VE-cadherin: vascular endothelial cadherin; KIM-1, kidney injury molecule-1; NPNT, nephronectin; 36B4, acidic ribosomal phosphoprotein P0.
