# Supplementary material for: Effects of crystalloid, hyper-oncotic albumin, and iso-oncotic albumin on lung and kidney damage in experimental acute lung injury
Source: Respir Res. 2019 Jul 16;20:155. doi: 10.1186/s12931-019-1115-x (PMC6636113; doi:10.1186/s12931-019-1115-x)
Supplement: Supplementary file 3 — Table S3. Arterial blood gas analysis. (DOCX 18 kb) [file 12931_2019_1115_MOESM3_ESM.docx]

**Supplemental Digital Content 3**

**Table 3 – Arterial blood gas analysis**

| **Parameter** | **Group** | **INITIAL** | **FINAL** | **Time Effect** | **Group Effect** | **Time *vs.* Group Effect** |
| --- | --- | --- | --- | --- | --- | --- |
|  |  |  |  |  |  |  |
| **Arterial pH** | RL | 7.34±0.07 | 7.31±0.09 | *p*=0.99 | *p*=0.16 | *p*=0.52 |
|  | 20%ALB | 7.38±0.07 | 7.38±0.06 |  |  |  |
|  | 5%ALB | 7.32±0.15 | 7.35±0.06 |  |  |  |
|  |  |  |  |  |  |  |
| **PaCO_2_** | RL | 27±7 | 23±6 | *p*=0.37 | *p*=0.03 | *p*=0.70 |
| **(mmHg)** | 20%ALB | 33±9 | 34±7 |  |  |  |
|  | 5%ALB | 39±21 | 32±13 |  |  |  |
|  |  |  |  |  |  |  |
| **PaO_2_/FiO_2_** | RL | 344±62 | 318±90 | *p*=0.41 | *p*=0.90 | *p*=0.09 |
| **(mmHg)** | 20%ALB | 335±116 | 330±73 |  |  |  |
|  | 5%ALB | 282±79 | 380±121 |  |  |  |
|  |  |  |  |  |  |  |
| **Anion gap** | RL | 22±4 | 26±7 | *p*=0.22 | *p*=0.003 | *p*=0.19 |
| **(mEq/L)** | 20%ALB | 21±5 | 20±4 ^*,#^ |  |  |  |
|  | 5%ALB | 27±1 | 26±2 |  |  |  |
|  |  |  |  |  |  |  |
| **Bicarbonate** | RL | 16.5±4.8 | 11.1±4.8† | *p*=0.003 | *p*<0.01 | *p*=0.02 |
| **(mEq/L)** | 20%ALB | 19.6±3.3 | 19.6±3.7^*^ |  |  |  |
|  | 5%ALB | 18.9±2.5 | 18.4±4.7^*^ |  |  |  |
|  |  |  |  |  |  |  |
| **Sodium** | RL | 146.0±4.4 | 151.5±4.1 |  |  |  |
| **(mEq/L)** | 20%ALB | 147.1±2.9 | 148.4±2.5 | *p*=0.37 | *p*=0.02 | *p*=0.007 |
|  | 5%ALB | 147.4±3.4 | 143.1±2.3 |  |  |  |

Data are shown as mean ± SD. Comparisons among groups at each time point were done by a mixed linear model based on a random intercept for each animal followed by Bonferroni’s test. RL: Ringer’s lactate; 20%ALB: 20% albumin; 5%ALB: 5% albumin; PaCO_2_: arterial partial pressure of carbon dioxide; PaO_2_: arterial partial pressure of oxygen; FiO_2_: fraction of inspired oxygen. * *vs* RL (*p*<0.05); # *vs* 5%ALB (*p*<0.05); †*vs* Initial within the respective group (*p*<0.05).
