# Supplementary material for: Effects of crystalloid, hyper-oncotic albumin, and iso-oncotic albumin on lung and kidney damage in experimental acute lung injury
Source: Respir Res. 2019 Jul 16;20:155. doi: 10.1186/s12931-019-1115-x (PMC6636113; doi:10.1186/s12931-019-1115-x)
Supplement: Supplementary file 4 — Table S4. Respiratory system and lung mechanical parameters (DOCX 21 kb) [file 12931_2019_1115_MOESM4_ESM.docx]

**Supplemental Digital Content 4**

**Supplemental Table 4: Respiratory system and lung mechanics parameters**

| Parameters | Groups | INITIAL | FINAL | Time Effect | Group Effect | Time *vs.* Group Effect |
| --- | --- | --- | --- | --- | --- | --- |
| **V_T_ (mL/kg)** |  |  |  | *p*=0.98 | *p*=0.17 | *p*=0.09 |
|  | RL | 6.5±0.8 | 6.9±0.9 |  |  |  |
|  | 20%ALB | 6.0±0.7 | 5.8±0.7 |  |  |  |
|  | 5%ALB | 5.7±1.7 | 5.5±1.1 |  |  |  |
| **RR (bpm)** |  |  |  | *p*=0.14 | *p*=0.49 | *p*=0.76 |
|  | RL | 80±8 | 77±12 |  |  |  |
|  | 20%ALB | 76±10 | 74.3±10 |  |  |  |
|  | 5%ALB | 70±18 | 69±12 |  |  |  |
| **PPlat,_RS_ (cmH_2_O)** |  |  |  | *p*=0.08 | *p*=0.88 | *p*=0.23 |
|  | RL | 13.4±1.8 | 16.7±3.2 |  |  |  |
|  | 20%ALB | 14.5±2.7 | 14.9±1.6 |  |  |  |
|  | 5%ALB | 14.9±3.7 | 15.4±1.2 |  |  |  |
| **PPlat,_L_ (cmH_2_O)** |  |  |  | *p*=0.24 | *p*=0.36 | *p*=0.40 |
|  | RL | 10.6±1.3 | 13.3±1.3 |  |  |  |
|  | 20%ALB | 12.6±2.8 | 12.8±1.8 |  |  |  |
|  | 5%ALB | 13.4±3.9 | 13.7±1.7 |  |  |  |
| **ΔP,_RS_ (cmH_2_O)** |  |  |  | *p*=0.13 | *p*=0.84 | *p*=0.44 |
|  | RL | 10.3±1.9 | 12.9±2.9 |  |  |  |
|  | 20%ALB | 11.4±2.8 | 11.9±1.6 |  |  |  |
|  | 5%ALB | 11.9±3.7 | 12.4±1.2 |  |  |  |
| **ΔP,_L_ (cmH_2_O)** |  |  |  | *p*=0.44 | *p*=0.15 | *p*=0.76 |
|  | RL | 7.6±1.4 | 9.2±3.4 |  |  |  |
|  | 20%ALB | 9.6±2.8 | 9.8±1.8 |  |  |  |
|  | 5%ALB | 10.4±3.9 | 10.7±1.7 |  |  |  |
| **Est,_RS_ (cmH_2_O/mL)** |  |  |  | *p*=0.37 | *p*=0.58 | *p*=0.50 |
|  | RL | 4.4±1.4 | 5.6±2.2 |  |  |  |
|  | 20%ALB | 4.5±0.8 | 4.8±1.2 |  |  |  |
|  | 5%ALB | 5.3±1.2 | 5.1±0.8 |  |  |  |
| **Est,_L_ (cmH_2_O/mL)** |  |  |  | *p*=0.36 | *p*=0.71 | *p*=0.39 |
|  | RL | 3.6±1.2 | 5.2±3.4 |  |  |  |
|  | 20%ALB | 4.0±0.7 | 4.2±1.3 |  |  |  |
|  | 5%ALB | 4.8±1.3 | 4.6±1.1 |  |  |  |
| **Energy (mJ)** |  |  |  | *p*=0.73 | *p*=0.19 | *p*=0.94 |
|  | RL | 0.8±0.1 | 0.9 ±0.6 |  |  |  |
|  | 20%ALB | 1.2±0.8 | 1.2±0.5 |  |  |  |
|  | 5%ALB | 1.1±0.5 | 1.2±0.1 |  |  |  |
| **Power (mJ/min)** |  |  |  | *p*=0.83 | *p*=0.44 | *p*=0.92 |
|  | RL | 65.4±5.6 | 75.3±49.8 |  |  |  |
|  | 20%ALB | 89.8±52.1 | 88.1±24.7 |  |  |  |
|  | 5%ALB | 82.7±11.5 | 82.7±11.5 |  |  |  |

Data are shown as mean ± SD. Comparisons among groups at each time point were done by a mixed linear model based on a random intercept for each animal, followed by Bonferroni’s test. RL: Ringer’s lactate; 20%ALB: 20% albumin; 5%ALB: 5% albumin; V_T_: tidal volume; RR: respiratory rate; PPlat,_RS_: respiratory system plateau pressure; PPlat,_L_: transpulmonary plateau pressure; ΔP,_RS_: respiratory system driving pressure; ΔP,_L_: transpulmonary driving pressure; Est,_RS_: respiratory system static elastance; Est,_L_: lung static elastance.
